# STTORM-CD low-demand and high-impact disaster monitoring onboard satellites using change detection

**DOI:** 10.1038/s41598-025-32598-3

**Published:** 2026-02-04

**Authors:** Jonáš Herec, Jan Sedmidubsky, Rado Pitoňák

**Affiliations:** 1 Masaryk University, Brno, Czech Republic; 2 Zaitra s.r.o., Brno, Czech Republic

**Keywords:** Environmental sciences, Natural hazards, Aerospace engineering, Computer science, Scientific data, Software

## Abstract

Satellite imagery can play a crucial role in disaster management, but critical images often take hours or even days to reach end-users, and upgrading hardware to improve transmission speed is prohibitively expensive for many small satellite missions. This article thus explores onboard change detection methods as a cost-effective alternative to reduce reaction time. Building on RaVAEn, we introduce STTORM-CD, a framework that combines a Variational Autoencoder (VAE) with a triplet loss, specifically designed for change detection. The triplet loss improves detection accuracy while maintaining the computational and storage efficiency of VAE, making it suitable for deployment on resource-constrained satellite hardware. To support training and evaluation, we present a new dataset, STTORM-CD-Floods, annotated with a custom strategy optimized for flood detection, along with new metrics, AURC and RDP, designed to address limitations of RaVAEn evaluation strategies, which are influenced more by dataset composition than model performance. Our experiments show that STTORM-CD outperforms existing flood detection methods, achieving an increase of  35 percentage points (pp) in custom AURC and standard AUPRC metrics against RaVAEn on the presented STTORM-CD-Floods dataset, while showing negligible changes in AURC (approximately -4 to +0.1 pp) for landslides and wildfires. This demonstrates that improvements on one disaster type do not necessarily compromise performance on others and highlights the potential for a universal and accurate real-time disaster detection system.

Between 2000 and 2023, 9,363 natural disasters were recorded, including 2,549 extreme weather events (such as hurricanes and tornadoes), 4,088 floods, and 304 wildfires. Floods alone resulted in 130,608 deaths and costs of 735.6 billion USD in damages^1^. While resilience to natural disasters is improving, particularly in developed nations^2^, worsening climate conditions remain a major concern. The latest Intergovernmental Panel on Climate Change (IPCC) report warns that climate change is driving a rise in the frequency and severity of natural disasters^3^. A comprehensive assessment of global climate change is beyond the scope of this study, but several examples highlight the growing concern. For example^4^, predicts a significant rise in flood frequency and magnitude over the next 50 years, with severity directly tied to the extent of temperature increase. Additionally, satellite data analysis from^5^ indicates that wildfires are becoming more frequent and intense, with six of the seven most extreme wildfire events in the past two decades occurring within the last six years.

Meanwhile, IT technologies can play a crucial role in every phase of disaster management, including prevention (e.g., vulnerability assessments), preparedness (e.g., disaster forecasting), response (e.g., detection of damaged areas), and recovery (e.g., assessing losses)^6^. We aim to contribute to this by improving the response and recovery phases through Earth monitoring from space. In urban disaster scenarios, satellite imagery can quickly provide critical information about the situation, enabling more effective aid distribution. In case of a disaster in a remote area, such as a wildfire in a secluded forest, satellite technology can help detect them and mitigate their spread/consequences. This approach not only has the potential to save lives and resources but could also play a crucial role in environmental protection.

However, accessing data from satellites remains challenging in terms of both resources and time. Satellites in LEO (Low Earth Orbit) can only transmit data to ground stations during specific windows when they pass overhead. Typically, this results in short downlink periods, conducted via low-bandwidth radio frequency communication and occurring a few times daily. So in practice, the images assessing the damages can arrive on the ground with an hourly to daily delay. This bottleneck can be addressed by integrating machine learning models onboard, allowing satellites to autonomously identify and prioritize critical data.

This is challenging due to the constrained power resources of satellites. Satellites commonly rely on solar energy for power and utilize well-established components that have been used for years. While this approach ensures safety and energy efficiency for satellites, it proves inadequate for most machine-learning models, as the computational power and storage size are limited. This is inflated for our use case, as detecting disasters is best framed as the detection of change. Otherwise, it is hard to distinguish between dirt and landslides, natural water objects and flooded areas, abandoned municipalities, and municipalities destroyed by hurricanes.

Change detection models typically take two images from the same location captured at different times, generating a map showing their differences^7^. As a result, these models are often twice the size of standard segmentation models and require significant computational resources and storage, as the image from before needs to be stored onboard. This was partially solved by recent research^8–10^, which proposed and tested change detection systems appropriate for limited computational resources onboard. However, this is insufficient because it does not solve the onboard storage limitations. These approaches still require storing the original pre-event images, which significantly reduces the area that a single satellite can monitor.

This challenge was addressed in RaVAEn^11^. Here, the change detection is done by comparing the cosine distance of embeddings from the Variational Autoencoder (VAE)^12^. Instead of saving the original image, only the embeddings need to be saved, effectively solving the limited storage limitations. However, RaVAEn’s performance is unpredictable, as it is trained in a self-supervised way. Thus, using the model, it can ignore floods and instead detect blooming fields as a significant change. However, this was not adequately demonstrated in the RaVAEn research due to the use of unsuitable metrics and a relatively weak baseline.

In this paper, we build on this research by leveraging RaVAEn and its compression capabilities, which, unlike traditional change detection networks, can be used to monitor entire continents, even with limited onboard storage. To overcome its limitations, we train the model with triplet loss, encouraging similar embeddings for harmless seasonal changes while enforcing strong separation for flood-related changes. Namely, our contributions are:

- Introducing STTORM-CD (“**S**iamese encoder **T**rained with **T**riplet loss for **O**nboard disaste**R** **M**onitoring by **C**hange **D**etection”).
- Introducing a new, simple yet effective annotation strategy for change detection.
- Creating a change detection dataset focused on floods – STTORM-CD-Floods.
- Introducing AURC and RDP, new metrics suitable for tile-wise change detection.
- Showing the potential of geo-indices differences as a baseline for change detection focused on natural disasters.
- Reevaluation of RaVAEn with the appropriate metrics and baseline.
- Quick survey into a currently unsolved and heavily related problem – onboard georeferencing.

If all insights from this study are met with a budget and international cooperation, a production-ready onboard change detection system for various disasters could be ready within a few years to save lives and resources worldwide.

## Background

### Smallsats

Smallsats weighing under 600 kg accounted for 96 % of launches in 2022, with a total of 2,478 deployed. Additionally, even smaller satellites (under 10 kg) are becoming increasingly common, making up 13 % of launches that year. While most smallsats belong to Starlink and OneWeb, a notable 10 % are dedicated to remote sensing^13^. The growing popularity of smaller spacecraft is primarily driven by cost reduction. Satellites can travel together on rideshares (e.g., aboard a SpaceX Falcon 9), with some no larger than a shoebox. The development and deployment of such satellites is relatively affordable, typically costing anywhere from hundreds of thousands to millions of euros.

However, this has significant implications. Spacecraft design follows the SWaP-C principle, where size, weight, power consumption, and cost must be carefully balanced for a specific mission. Lowering costs almost inevitably requires compromises in other SWaP-C elements. As a result, downlink rates heavily depend on the mission budget and can vary widely, typically ranging from 0.5 to 12 MBps^14^, excluding outliers. Communication is primarily conducted via radio frequency, and achieving higher downlink rates requires larger, more powerful antennas, which in turn increase weight, power consumption, and, consequently, cost. Additionally, satellites can only communicate with a limited number of ground stations a few times per day and for short time windows. Many satellite operators also rely on external ground station networks, which typically operate on a pay-per-pass basis.

This can be a challenge, as satellite imagery requires significant storage space. Unlike conventional three-channel (RGB) images, remote sensing cameras can capture light across dozens or even hundreds of spectral bands, depending on the mission. Typically, this falls into either multispectral imaging, with around 10 channels (e.g., Landsat 8, Sentinel-2), or hyperspectral imaging, which can exceed 200 channels (e.g., PRISMA, EMIT, EnMAP). In addition to spectral composition, satellite images also vary in ground sampling distance (GSD). For example, a GSD of 10 meters means that each pixel represents a 10$\times$10 meter area on Earth’s surface. In recent years, satellites have achieved GSDs finer than 1 meter, further increasing data volume and storage demands.

This underscores the need to minimize data size during transmission, driving the trend of embedding machine learning algorithms in satellites to autonomously filter and prioritize data. However, the computational resources of satellites are highly limited. Data processing units (DPU) must withstand launch stresses, prolonged radiation exposure, and operate on limited solar power, making development expensive and time-consuming.

DPUs can use GPUs like the Jetson Orin Nano or Xavier NX, but these increase power consumption and remain largely untested in space. Since ML filtering is a non-critical payload component meant to save time and costs, increasing spacecraft risk and power demands for better model performance contradicts this goal.

A common approach is thus using low-power CPUs or CPU-FPGA combinations, which are space-qualified through years of use. Mid-range examples include the Xiphos Q7S CDH with an AMD Xilinx Zynq 7000 series (up to dual-core ARM Cortex-A9 at 1GHz), while higher-end options like the Xiphos Q8 use the AMD-Xilinx Zynq Ultrascale+ MPSOC (FPGA and Quad-core ARM Cortex-A53 at 1.5GHz). Though powerful for space applications, they lag behind terrestrial GPUs, requiring ML models to be compact and efficient for CPU execution or small enough for deployment on an FPGA.

Finally, storage is also a key factor in change detection. Satellites use storage solutions, highly dependent on the mission budget, ranging from lightweight SD cards or eMMC to heavier SSDs, with capacities typically ranging from tens to hundreds of gigabytes, and in some expensive exceptions, a few terabytes.

To conclude, to not incur additional costs, the change detection system must be computationally and storage efficient. If it achieves this, it could be the only way to significantly reduce the downlink time of crucial imagery while keeping the costs reasonable.

### Related works

A common architectural approach in change detection involves employing an encoder-decoder model with skip connections, such as the U-net model^15^. Unlike the original U-net, which processes a single image to produce a segmentation map, the change detection variant takes two images as input and generates a change map^7^. These images can be concatenated and fed into the model simultaneously or processed separately through distinct encoders. The output of the encoder(s) is then forwarded to the decoder, which generates the change map. The most recent SoTA change detection models often have a transformer-based architecture with various modifications. Still, they can be outperformed by the mentioned simple U-net-like architecture under some conditions^7^. However, for onboard use, they all would have to be extensively pruned.

Recent research has focused on such tasks, using methods like knowledge distillation^8^, where a compact model learns from a larger one, or unsupervised approaches^10^, where change detection is performed through subtraction and other operations on feature maps from pretrained convolutional layers of an encoder. Another study^9^ uses a two-phase approach, first classifying the image as containing change or not, and only performing pixel-wise change detection if a change is detected.

Although these studies present interesting findings and can operate onboard, we argue that their practical application potential is limited. Firstly, they primarily use common change detection datasets that monitor gradual changes in urban areas^16,17^, which are less relevant for onboard applications, as there is no pressing need to deliver these changes rapidly. But mainly, these approaches use original uncompressed images, meaning that onboard storage would need to accommodate the full-sized images. While lossless onboard compression could reduce their size (we can expect around a 1:4 compression ratio^18^), this is likely still insufficient, as the storage limitations of most smallsats would restrict monitoring to small areas only.

These limitations were addressed in the RaVAEn research^11^. It employs a Variational Autoencoder (VAE)^11^, a symmetric network structure where the encoder progressively reduces layer size, culminating in a bottleneck (latent space) that follows a Gaussian distribution. The decoder then gradually expands the layers back to the original size. Since the input and label are the same image, the network is trained to efficiently compress the input image into the latent space – thus creating the embedding.

When the satellite takes a new picture, the VAE encodes the picture into an embedding. Then, the cosine distance of this embedding and the embedding of the picture of the same area from the past is computed. If the distance is high, the image is probably different, which signals a potential disaster. By prioritizing images based on their cosine distance, the most suspicious ones can be transmitted first. This approach offers three key advantages over the other mentioned approaches.

Firstly, its potential for compression is immense. The current compression rate of RaVAEn is 1:40, and this could be significantly increased by finding an optimal combination of input size and latent space size, using half-precision floats, quantization, or a binary latent space. Secondly, it is more efficient. Each image has to be processed into an embedding only once, and then a computationally cheap cosine distance can be used to calculate the change between the embeddings. Thirdly, due to the self-supervised way of learning, it can learn to capture essential image characteristics within the embedding without needing costly labeled data.

These advantages suggest that developing the change detection system based on the VAE can create a computationally and storage-efficient algorithm that can be developed without extensive cost. However, there is also an important limitation. The features are learned to be extracted in such a way that they follow a Gaussian distribution and can subsequently be decoded. However, the distances between embeddings may not hold meaning for the specific use cases. For instance, the model may learn to create a significant distance for areas with and without snow, or regions with and without cloud shadows, and it may ignore a flooded area. However, this could be potentially solved via Siamese networks, specifically designed to learn meaningful distances between embeddings.

Classical Siamese networks consist solely of an encoder, which transforms input into embeddings. These networks are trained using pairs or triplets of data, where they learn to move the distance between embeddings of similar inputs closer and move the distance between embeddings of dissimilar inputs further^19,20^. Due to their way of training, they do not tend to overfit, and it has been demonstrated that they are capable of few-shot learning^21–23^, which is ideal for disaster scenarios where the creation of labels can be time and resource-consuming. This is done mainly via two loss functions – contrastive and triplet loss. For the disaster use case, triplet loss is more appropriate, as each training example teaches the network to recognize disasters while disregarding seasonal changes.

## Methodology

### Data

All data used in this study were taken by Sentinel 2^24^. Sentinel-2 consists of two satellites, launched in 2015 and 2017, and is operated by the European Space Agency under the EU Commission’s Copernicus Programme. They have the same instruments and orbit. However, they are apart by 180 degrees of longitude. This allows for a short revisit rate ($\le$ 5 days), which is ideal for change detection. Please note that the third satellite (Sentinel-2C) was launched in 2024^25^, further reducing the revisit rate. However, this study utilizes data exclusively from the earlier satellites, Sentinel-2A and Sentinel-2B. They both have multispectral instruments (MSI) capable of measuring 13 channels (bands) from 423 to 2370 nm, the GSD (ground sampling distance) of the channels varies from 10m (RGB+NIR) through 20m (6 channels in SWIR) to 60m. Following RaVAEn, the three 60 m channels were excluded, and all images used in this work were processed at the L1C level. In addition to the original channels, all used data also has a channel with a cloud mask generated by s2cloudless^26^ used for excluding cloudy data.

**Fig. 1:**
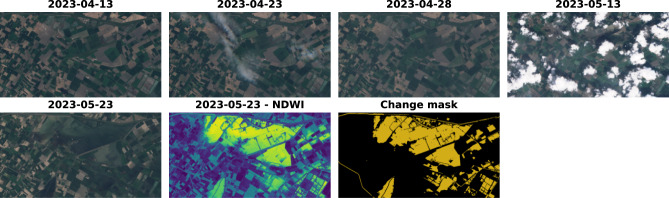
Annotation set and resulting mask – Italy.

#### Geo-indices

Multispectral instruments like Sentinel 2 have various benefits. Separating the light into multiple channels can help monitor the Earth, mainly because Earth features like vegetation or water have specific reflectance patterns (concerning wavelength). These differences can be utilized via geo-indices, which we propose to use as a change detection baseline. For example, to detect a change (flood), the geo-index extracting water can be used; the geo-index values of the current image can be compared against the geo-index values of the image from the past. If the area is not flooded or it is a natural water object, the values should be similar. If the area is flooded, the geo-index values in the current image should be higher. In this research, three geo-indices will be used as defined in Eqs. 1, 2, 3: Normalized Difference Water Index (NDWI)^27^, Normalized Difference Vegetation Index (NDVI)^28^, and Normalized Burn Ratio (NBR)^29^. Here, the B03, B04, B08, and B12 refer to the indices/names of Sentinel-2 bands (channels), while GREEN, RED, NIR (Near Infrared), and SWIR (Short-Wave Infrared) represent the corresponding portions of the electromagnetic spectrum they measure:

1 $$\begin{aligned} NDWI= & \frac{GREEN-NIR}{GREEN+NIR} = \frac{B03-B08}{B03+B08}\end{aligned}$$

2 $$\begin{aligned} NDVI= & \frac{NIR-RED}{NIR+RED} = \frac{B08-B04}{B08+B04}\end{aligned}$$

3 $$\begin{aligned} NBR= & \frac{NIR-SWIR}{NIR+SWIR} = \frac{B08-B12}{B08+B12} \end{aligned}$$

**Fig. 2:**
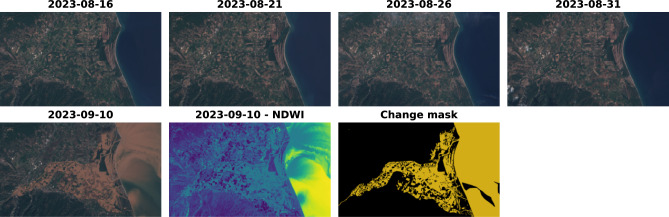
Annotation set and resulting mask – Greece.

### Datasets: RaVAEn-Floods and proposed STTORM-CD-Floods

The flood datasets used have a total of 20 *time series* with each time series consisting of 5 captured pictures – 4 pictures taken before the flood and 1 picture taken after the flood. The training, validation, and test sets contain 12, 4, and 4 time series, respectively. In this paper, we create new precise annotations of the flooded areas, which are used for defining the training and test sets, referred to as STTORM-CD-Floods. The validation examples are taken from the RaVAEn-Floods dataset. The scene resolutions in the STTORM-CD-Floods dataset range from approximately 1768$\times$933 to 1834$\times$1073, while those in the RaVAEn-Floods dataset range from 1042$\times$859 to 2674$\times$2194. The scenes from the train set of STTORM-CD-Floods and the RaVAEn-Floods were captured in Southern Europe (Italy, Greece, Spain, and France), while the scenes from the test set of STTORM-CD-Floods were sourced from various locations worldwide, namely Laos, Brazil, Niger, and Germany. This diverse selection allows for the validation of how well the model trained and validated on a small set of pictures from nearby regions can generalize to pictures from different parts of the world.

**Fig. 3:**
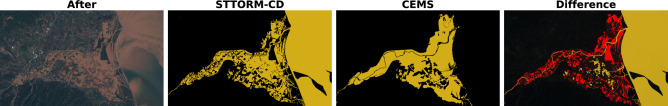
Comparison of annotation quality between the STTORM-CD mask and the CEMS mask for Event ESMR692 on the same date. Due to tight delivery timelines, CEMS products can be coarse and are primarily accurate near flood boundaries, which can lead to a notable number of false positive pixels. Additionally, our annotations include flood-related changes, such as the ocean becoming muddy, reflecting a difference in annotation approaches rather than an error.

#### Annotation strategy

The data was annotated using the Computer Vision Annotation Tool (CVAT)^30^. During the annotation, the images taken before the flood, after the flood, and the NDWI of the post-flood image were all available. Specifically, all these images were uploaded into a single CVAT task for each time series. In this setup, the annotator can zoom in and navigate within the spatial dimension of the time series while switching between the images. This approach helps significantly in two ways: First, it allows the annotator to scan for small changes that may not be immediately visible without having a direct, zoomed comparison against the pre-flood image; and second, it provides a broader context when annotating uncertain areas. The NDWI was particularly helpful for distinguishing between dark water bodies and grass fields, as seen in Fig. 1. However, it served only as a support, as it is unreliable if the water is muddy, as seen in Fig. 2. The annotations are almost pixel-perfect, as for annotating delicate areas, a brush with a width of 2 pixels was utilized. The quality of annotation is shown in Fig. 3, where it is compared to the expert-labeled mask from Copernicus Emergency Management Service (CEMS)^31^. After the final image was annotated, all annotations were reviewed and refined by the annotator and further checked by two remote sensing experts. We want to clarify that there was only one annotator, so this final review ensured consistency across the entire dataset, as the initial annotations may have been of lower quality compared to the final ones, due to the natural learning curve of the annotator who gradually became an expert.

### Formalization

**Fig. 4:**
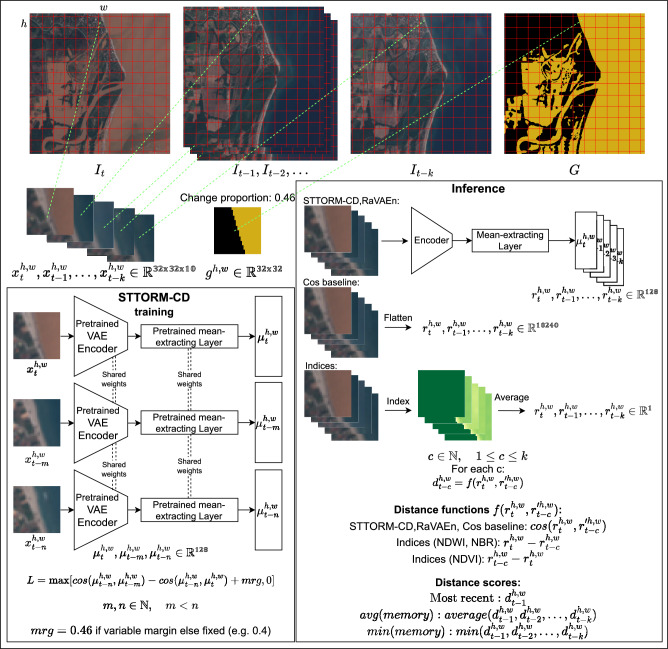
Illustration of STTORM-CD approach and tile-wise change detection.

We present a formalization of the proposed change detection approach to make it clearly comprehensible. We have a satellite image $I_t$ taken after the disaster at time *t* and *k* images $I_{t-1}, I_{t-2}, \ldots , I_{t-k}$ taken before the disaster at times $t-1,t-1,\ldots , t-k$, i.e., these images are taken at the same location at different times. We also have a ground truth mask *G* segmenting all changed pixels (e.g., flooded) in the time series of such images, visualized in yellow in Fig. 4. Then, we slide the 32x32px window across each image, and at each location *h*, *w*, where *h* represents the height and *w* represents the width of the window’s position within the image, we extract image *tiles* $x^{h,w}$, resulting in a time series of tiles $x_{t}^{h,w}, x_{t-1}^{h,w},\ldots ,x_{t-k}^{h,w}$ and a corresponding ground truth tile $g^{h,w}$. The window is slid by 32 pixels in each step (stride), so the extracted tiles do not overlap. Each tile is then processed into a comparable representation $r_{t}^{h,w}$. In the case of the RaVAEn^11^ models, this is the vector of VAE means $\mu _{t-k}^{h,w}$ from the latent space, as used in their article. This is the same for our approach (STTORM-CD model), as it is the same model, but fine-tuned via the triplet loss. In the case of the cosine baseline, this is a flattened original tile. In the case of the NDWI, NDVI, and NBR geo-indices, this is the average pixel value for a tile processed by the geo-index.

We apply the change detection function *f*() on these representations. In the case of the cosine baseline, RaVAEn models, and the STTORM-CD models, this is cosine distance – e.g., $cos(r_{t}^{h,w}, r_{t-k}'^{h,w})$ (the cosine distance was selected against alternatives such as the Euclidean distance based on RaVAEn experiments, where it was the superior performer). In the case of the geo-indices, this is a simple subtraction. For NBR and NDWI, this is $r_{t}^{h,w}-r_{t-k}^{h,w}$, as higher values signal more water or burnt area, meaning that if we have more of it in the picture after, there is probably a disaster. Whereas in the case of the NDVI, this is reversed, $r_{t-k}^{h,w}-r_{t}^{h,w}$ as higher values signal healthy vegetation, and after the disaster, there is probably less healthy vegetation. In summary, doing the distance function *f*() yields *k* predictions of change $d_{t-1}^{h,w}, d_{t-2}^{h,w},\ldots ,d_{t-k}^{h,w}$. This is because we apply the function on each combination of representation of the image before $r_{t-1}^{h,w}, r_{t-2}^{h,w},\ldots ,r_{t-k}^{h,w}$ and representation of the image after $r_{t}^{h,w}$.

The change predictions can be then utilized in 3 different ways: We take (1) only the most recent change prediction $d_{t-1}^{h,w}$, (2) the minimum $min\{d_{t-1}^{h,w}, d_{t-2}^{h,w},\ldots ,d_{t-k}^{h,w}\}$ of the change predictions denoted as *min*(*memory*) in the results, which enables ignoring small fluctuations, or (3) the average $avg\{d_{t-1}^{h,w}, d_{t-2}^{h,w},\ldots ,d_{t-k}^{h,w}\}$ of the change predictions denoted as *avg*(*memory*) in the results, to get a comprehensive and history-attentuated final prediction. The final prediction can then be compared against the sum of changed pixels in ground truth tile $g^{h,w}$.

### Preprocessing

The image data are in digital number format, which means that they are composed of 16-bit integers with meaningful values ranging from 1 to 65,535, while 0 is reserved for missing values. To ensure utilizing the full potential of RaVAEn pre-trained network weights, the preprocessing was done in the same way as in RaVAEn. This means transforming values using natural logarithm first, followed by scaling approximately to the interval $[-1,1]$ based on the RaVAEn observed maximum and minimum logarithm values. However, to prevent numerical instability, the zero values were clipped to 1 before taking the logarithm. For training, validation, and testing, the data was divided into tiles using a sliding window of size 32$\times$32. This matches the tile size used in RaVAEn, which is also essential for leveraging their pretrained models. The window was moved by 32 pixels (stride) for testing and validation to ensure non-overlapping windows. However, for training, the stride was kept as a hyperparameter.

After tiling, filtering was applied. The RaVAEn validation scenes contain NaNs on the sides of the picture, as the scene is often rotated. Tiles containing NaNs or cloud coverage greater than 25 % were excluded from training and evaluation. It is important to remember that the data are in time-series format and that there are 5 tiles for each tile location (i.e., 1 post-disaster tile and 4 pre-disaster tiles). The post-disaster tile is compared against the pre-disaster tiles during training and evaluation. So if the post-disaster tile has cloud coverage larger than 25 %, the whole tile time series is discarded. Whereas if a pre-disaster tile has cloud coverage larger than 25 %, only this tile is discarded. If all pre-disaster tiles are removed, the whole tile time series is discarded. Tiling the scenes with a stride of 32 and filtering out cloudy tiles and tiles with NaNs, resulted in the training, validation, and testing datasets containing 16,442, 11,253, and 6,678 time series of tiles.

### STTORM-CD

We propose the STTORM-CD approach – “**S**iamese encoder **T**rained with **T**riplet loss for **O**nboard disaste**R** **M**onitoring by **C**hange **D**etection”. In particular, we take an encoder from RaVAEn, which is a VAE trained in a self-supervised way on the WorldFloods dataset^32^, consider it a siamese network, and use triplet loss alongside a dataset of small size to fine-tune it. The underlying idea is that the structure of the data is learned in the more massive and practically costless VAE training, and then the few high-quality labeled images are used to guide the network on what specific features to identify. The VAE encoder creates both means and log variances. For the change prediction, only cosine distances of means are utilized, so besides the decoder, we have also discarded the fully connected layer, utilized for transforming the features into log variances. We have fine-tuned all 3 models proposed in RaVAEn. We have not changed the architecture, but we have completely changed the training process. The training is done in triplets as defined in Eq. 4:

4 $$\begin{aligned} L(\mu _{t-n}^{h,w}, \mu _{t-m}^{h,w}, \mu _{t}^{h,w}) = \max \{cos(\mu _{t-n}^{h,w}, \mu _{t-m}^{h,w}) - cos(\mu _{t-n}^{h,w}, \mu _{t}^{h,w}) + \text {margin}, 0\},\quad n, m \in \mathbb {N}, \quad \quad n > m \end{aligned}$$

To effectively distinguish flooded areas from non-flooded ones, the network must ignore seasonal and other irrelevant changes while ensuring that flood-related changes are correctly identified. The triplet loss function is well-suited for this purpose, as it encourages similar representations for pre-flood images while separating the representations of flooded images from them.

In this framework, an image tile captured well before the flood serves as an anchor. A positive example is an image tile captured closer to the flood but still before its occurrence, ensuring an inclusion of irrelevant seasonal changes. A negative example corresponds to an image tile taken after the flood, where significant changes are expected. The function optimizes the representation space so that the distance between the anchor and the positive example is separated by at least a specified margin from the distance between the anchor and the negative example.

Formally, considering the notation defined earlier, the approach is structured as follows. The distance function is given by cosine distance, denoted as $\cos ()$. The negative example $\mu _{t}^{h,w}$ is a vector of VAE means derived from a tile captured after the flood. The positive example $\mu _{t-m}^{h,w}$ is the same vector but obtained from a tile captured before the flood, and the anchor $\mu _{t-n}^{h,w}$ is obtained from a tile taken even further before the flood.

The margin in the triplet loss was set as a variable instead of a fixed constant to utilize all tiles and not only the small portion of tiles that were changed. This means that triplets where the negative tile has 53 % of pixels changed, had a margin of 0.53, triplets with 0 % of changed pixels had a margin of 0.0, etc. However, this was kept as a hyperparameter, and a traditional fixed margin was also used. In the case of a fixed margin, only triplets with a change proportion bigger than the margin were used. So, if the margin was fixed at 0.5, only triplets, where the negative tile had more than 50 % of pixels changed, were used. Figure 4 illustrates the overall process.

In each time series, multiple possible combinations of triplets exist. Even though there is only one image after the flood, there are 4 images before. Every possible combination of anchor-positive-negative was extracted into training triplets so the models could learn to ignore changes occurring with time. This resulted in app. 63.5k of training triplets if the margin was a variable. When using a fixed margin of 0.5, the training dataset consisted of 15.6k triplets.

### Compression

The network input is a 32x32x10 image, originally stored in 16-bit integer format, with a total size of 20,480 bytes (calculated by multiplying the number of values by the bit depth, ignoring structural overhead). In comparison, each embedding consists of 128 floats (32-bit), totaling 512 bytes. When using indices, only the average pixel value needs to be stored, represented by a single 32-bit float, resulting in 4 bytes per tile. The corresponding compression ratios and practical storage requirements for different area sizes are summarized in Table 1. Clearly, monitoring large areas within limited storage space becomes unfeasible without substantial compression, such as through embeddings or indices.

**Table 1:** Storage requirements for different representations per unit area. Computed with the world partitioned into 32x32x10 uint16 images with 10 m GSD. The structural overhead is ignored in the computation. The 1:4 compression ratio of the lossless compression is approximated, based on^18^.

Method	1 km²	Europe (without Russia) (6,184,800 km$^2$)	World (132,025,199 km$^2$)	Compression ratio
Original image (Uncompressed)	195.31 kB	1,151.99 GB	24,591.29 GB	1:1
Original image (Lossless compression)	48.82 kB	287.95 GB	6,146.87 GB	1:4
STTORM-CD / RaVAEn embeddings	4.88 kB	28.78 GB	614.43 GB	1:40
Indices	0.03 kB	0.17 GB	3.77 GB	1:5120

## Metrics

Comprehending the RaVAEn evaluation method, its flaws, and our proposed metrics is neither a quick nor an intuitive task. Therefore, a detailed explanation is provided in the Supplementary Information (see the “Metrics” section, with Figures S1, S2 and S3), while a brief overview is presented here.

In RaVAEn evaluation, heatmaps are generated from the change predictions and compared to the ground-truth masks using the Area Under the Precision-Recall Curve (AUPRC). However, since the change prediction is a single value for an entire 32x32px tile, this method requires approximating the per-pixel prediction of change by assigning the same change prediction to each pixel in the tile. This approach strongly favors scenarios with a higher proportion of changed pixels within tiles (e.g., wildfires), as recalling tiles with only some percentage of change significantly decreases precision. Since the metric is significantly influenced by the change distribution within the tiles, it tends to diminish differences between the models. Additionally, the AUPRC was incorrectly computed for each image individually and then averaged, instead of being computed across the entire dataset using only a one set of thresholds.

Since no existing metric seems to be appropriate, we have decided to propose two custom metrics to better reflect real-life performance: Area Under Recall Curve (AURC) and Required Downlink Percentage (RDP). As previously discussed, the change predictions can be used to prioritize tile ordering, ensuring that the most affected areas are delivered first, followed by the peripheries of the catastrophe. This principle is captured in the AURC, which evaluates how well the system’s ordering of tiles aligns with the ground truth. While the underlying principle is similar to weighted correlation, what AURC measures is more directly relevant to our task and has a straightforward interpretation: for different amounts of data that the ground station can download in a single pass, what percentage of changed pixels that could have been retrieved with a perfect system were actually retrieved? Since AURC focuses on the most affected areas and the top percentages of downlinked data, we propose a complementary metric that considers the entire dataset. This is the RDP metric, which is simple – it represents the percentage of data that must be downlinked to deliver all changed tiles (defined as $>5\,\%$ of pixels changed in the corresponding ground truth tile).

## Results

We first report the results for the test set of the introduced STTORM-CD-Floods dataset and then revisit the RaVAEn datasets using the proposed metrics, geo-indices as baselines, and the STTORM-CD models. All STTORM-CD models were trained on an Nvidia GeForce RTX 3090. We have conducted an ablation study comparing the two proposed margin strategies. For each model size, the top-performing models trained under each margin strategy were evaluated across all datasets and metrics. In brief, while performance on flood datasets was generally similar across strategies, the variable margin approach achieved notably higher scores for other disaster types, likely due to its inclusion of non-flooded images. Consequently, all subsequent STTORM-CD model results are reported for models trained using the variable margin. For the ablation study in greater detail, alongside the hyperparameters used to obtain the best models, refer to the Supplementary Information (see the “Training details and ablation study” section, with Tables S1 and S2).

All STTORM-CD models are ideal for space hardware as they are quite small, with the model sizes in ONNX format being 0.62 MB, 1.43 MB, and 2.99 MB for the small, medium, and large models, respectively. Their runtime and throughput when deployed on a CPU with Apache TVM^33^ can be seen in Table 2. These measurements were conducted on the Xiphos Q8, a representative high-end satellite board with over 100 units deployed in space.

**Table 2:** A comparison of the STTORM-CD models. The mean cosine distance was computed using five embeddings from the history. The statistics are based on 10,000 measurements for a single 32$\times$32x10 tile. Throughput was calculated for input data in the form of 16-bit integers, which is commonly the format of raw satellite imagery. The results are presented in the format “minimum-maximum, $\mu$**: mean**”. The measurements were conducted on an ARM Cortex A53, utilizing all four 1.5GHz cores on the Xiphos Q8 board.

Size	Params (mil.) $\downarrow$	Model Only		Model + Mean Cosine Distance	
		Runtime (ms) $\downarrow$	Throughput (Mbps) $\uparrow$	Runtime (ms) $\downarrow$	Throughput (Mbps) $\uparrow$
small	0.156	1.35-89.8, $\mu$**: 2.56**	1.83-121.26, $\mu$**: 91.84**	2-198.37, $\mu$**: 3.43**	0.83-81.87, $\mu$**: 63.93**
medium	0.358	2.47-193.73, $\mu$**: 3.98**	0.85-66.36, $\mu$**: 51.96**	3.15-94.32, $\mu$**: 4.88**	1.74-52.1, $\mu$**: 41.14**
large	0.746	5.25-93.34, $\mu$**: 7.94**	1.76-31.22, $\mu$**: 24.31**	5.93-171.42, $\mu$**: 8.87**	0.96-27.62, $\mu$**: 21.48**

Table 3 shows results on the test set of the STTORM-CD-Floods dataset using both proposed metrics. The STTORM-CD models performed the best, overcoming RaVAen^11^ AURC by approximately 30 percentage points (pp) for all usages of memory. The second best method is the NDWI, worse than the STTORM-CD models by less than 10 pp. Third is the cosine distance of the original tiles, and distinctively worst are the RaVAEn models. STTORM-CD models performed better by a small margin ( 2–3 pp) when *avg*(*memory*) was used. This makes sense as averaging captures a comprehensive overview of past data, which can increase performance. It may also raise the risk of false positives due to seasonal changes, but the model is trained to filter them out. Meanwhile, for the cosine baseline and the RaVAEn models, the best results were obtained, also by a small margin ( 2–4 pp), by using the *min*(*memory*), which allows ignoring some of the seasonal changes at the cost of losing some information.

For the RDP, the best results across memory utilization strategies were also scored by the STTORM-CD models; however, for the medium-sized model, the better score was yielded by the RaVAEn for all memory strategies, with differences ranging from marginal <1 pp to more obvious  8 pp. It is possible that this model converged to a local minimum where the AURC, prioritizing rapid detection of as much change as possible, was optimal, but this came at the expense of precision on less changed tiles, lowering the RDP. With the best RDP configuration (small STTORM-CD model and *min*(*memory*) strategy), 31.55% of the downlink could be saved. The second best is the cosine baseline, except when using *avg*(*memory*), where the RaVAEn models perform better. The NDWI has the distinctly worst results. You can see why, in further visualization (Fig. 6): the distinction between detected water bodies and other parts of the images is quite small. So, it is predictable that changed tiles with a smaller change proportion will be mixed up with the unchanged tiles. For RDP, the best scores for all methods are obtained by using *min*(*memory*), probably due to the increased resistance against the false positives.

**Table 3:** Comparison of approaches’ performance on the test set from the STTORM-CD-Floods dataset. For RDP interpretation, it is important to mention that the total number of tiles in this dataset is 6,678, and 22.34 % of them contain the defined change ($>5\,\%$ of pixels changed). The “Most recent” column represents results from comparing the changed tile with only the most recent non-cloudy tile. In other columns, all non-cloudy tiles from memory were used to derive the final change predictions using the *min*() or *average*() function. Highlighted are the best scores for STTORM-CD models and RaVAEn models for easy comparison.

Method	AURC [%] $\uparrow$			RDP [%] $\downarrow$		
	Most recent	*min*(memory)	*avg*(memory)	Most recent	*min*(memory)	*avg*(memory)
Geo-Index	77.67	75.55	77.25	99.73	99.60	99.36
Cosine baseline	62.96	75.07	73.43	81.70	78.12	83.12
RaVAEn – small	47.00	51.96	50.00	80.70	**78.15**	81.87
RaVAEn – medium	**50.19**	**54.43**	**50.50**	83.36	80.95	**80.92**
RaVAEn – large	47.81	52.33	48.54	**80.59**	78.56	84.10
STTORM-CD – small	81.71	83.18	86.11	**72.58**	**68.45**	80.44
STTORM-CD – medium	**82.72**	**84.22**	**86.54**	85.91	81.49	88.26
STTORM-CD – large	79.87	81.92	83.99	75.89	71.70	**76.43**

**Fig. 5:**
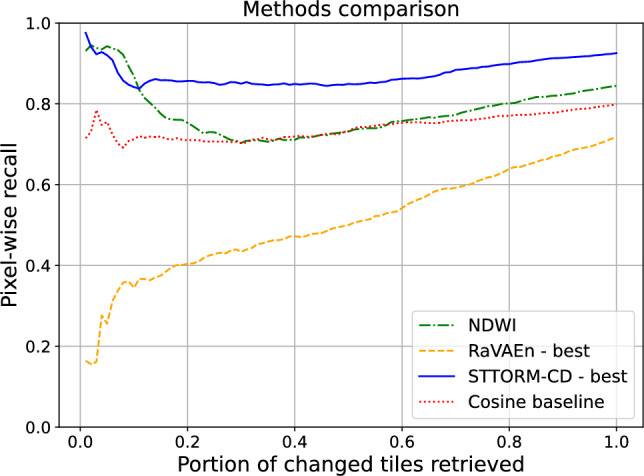
Recall curve visualization on the test set of STTORM-CD-Floods dataset. For almost all data volumes downlinked, the STTORM-CD delivered the most change, except when downlinking only the very top portion ( 2-10 %), where the NDWI was slightly better. The best STTORM-CD model is the medium-sized one, and the best RaVAEn model is also the medium-sized one. All presented methods derived their score using *avg*(*memory*).

The visualization in Fig. 5 shows the model performance at various retrieved tile portions. For most of the volume of the downlinked data, the STTORM-CD model performs the best. However, there is a region roughly between the top 2–10 % of the changed tiles retrieved where the NDWI performs better. It can also be seen that the RaVAEn model has trouble retrieving the most changed (top 10 %) tiles, resulting in retrieving only approximately 20–40 % of what it could have retrieved.

**Fig. 6:**
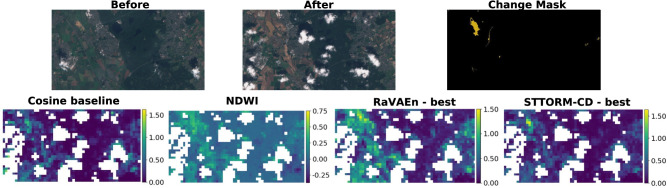
Germany – Visualization of method predictions. All predictions are quite noisy, except for the STTORM-CD model and Cosine baseline, but only STTORM-CD ignores the cloud shadows and confidently flags the flooded region. The best STTORM-CD model is the medium-sized one, and the best RaVAEn model is also the medium-sized one. All presented methods derived their score using *avg*(*memory*). The scale was set for each method using the *min*() and *max*() from the whole dataset to see an accurate performance.

A flood in Germany from the test dataset, visualized in Fig. 6, shows quite a distinction between models’ performance. All model predictions are quite noisy, except for the STTORM-CD model, which confidently predicts the biggest change for the most changed region in the picture. The cosine baseline predictions are probably the second best as they unconfidently predict change for the flooded region, but they also do so for cloud shadows.

**Table 4:** Reevaluating RaVAEn. The final change predictions were derived by using $\min ()$ on the change predictions from the memory. In RaVAEn-Floods, the geo-index used was NDWI; in RaVAEn-Wildfires, the NBR; and in RaVAEn-Landslides and RaVAEn-Hurricanes, NDVI. Highlighted are the best scores for STTORM-CD and RaVAEn models for each metric. All values of the evaluation metrics are reported as percentages.

Dataset	RaVAEn-Landslides		RaVAEn-Wildfires		RaVAEn-Hurricanes		RaVAEn-Floods	
Tiles count	626		27865		11773		11253	
Changed tiles [%]	23.64		57.89		27.57		21.70	
Metric	AURC $\uparrow$	RDP $\downarrow$	AURC $\uparrow$	RDP $\downarrow$	AURC $\uparrow$	RDP $\downarrow$	AURC $\uparrow$	RDP $\downarrow$
Geo-index	85.49	96.49	95.83	100.00	85.09	99.99	67.40	99.99
Cosine baseline	75.71	62.46	91.99	99.67	69.88	98.29	73.41	98.20
RaVAEn – small	87.03	**47.60**	91.87	98.74	**80.15**	**97.88**	**76.46**	95.84
RaVAEn – medium	**88.20**	48.56	91.98	**98.26**	78.80	98.68	75.59	94.67
RaVAEn – large	87.73	50.64	**92.29**	99.02	79.16	97.99	74.07	**94.11**
STTORM-CD – small	82.81	55.27	89.09	99.15	69.79	98.52	85.68*	87.45*
STTORM-CD – medium	86.71	**46.17**	87.97	**98.61**	73.51	96.49	85.46*	89.31*
STTORM-CD – large	**87.74**	52.08	**92.38**	99.47	**74.73**	**94.89**	84.50*	95.19*

*Used as a validation dataset.

The results from the RaVAEn datasets are shown in Table 4. STTORM-CD models perform best on the RaVAEn-Floods dataset, with the AURC for each model size exceeding its RaVAEn counterpart by  10 pp. This is expected, as the STTORM-CD models were fine-tuned for floods, and RaVAEn-Floods was used as the validation dataset. For RaVAEn-Hurricanes, the AURC differences are noticeable: slight drops of  5 pp occur for the medium and large models, while the smallest model shows a substantial drop of  10.5 pp. For other disasters, the changes are less noticeable. For RaVAEn-Landslides, the best-performing method is the RaVAEn medium model, but the difference from the best STTORM-CD model is minimal ( 0.5 AURC pp). Retraining RaVAEn models decreased AURC slightly for the small and medium models ( 1.5–4.2 pp) and led to a marginal increase for the large model (+0.01 pp). For RaVAEn-Wildfires, retraining the RaVAEn models on STTORM-CD also slightly decreased AURC for the small and medium models ( 3–4 pp) while producing a negligible increase for the large model ( 0.09 pp). For RaVAEn-Hurricanes and RaVAEn-Wildfires, the geo-indices performed best, outperforming the second-best method by approximately 3-–5 pp AURC. Except for RaVAEn-Floods, they consistently surpassed the cosine baseline, though by varying amounts ( 4–10 pp).

On the other hand, for the RDP metric, the geo-indices perform the worst across all datasets, as observed also for the STTORM-CD-Floods test set. For the RaVAEn-Floods dataset, as expected, the best result is achieved by the STTORM-CD model, although there is variability between model sizes, with a  8 pp difference between the small and large models. For RaVAEn-Hurricanes, the best result is also achieved by the STTORM-CD model, but only with a small difference compared to the best RaVAEn model ( 3 pp). A relatively large image in the RaVAEn-Hurricanes dataset, showing flooding caused by Hurricane Harvey, likely contributed to this outcome. However, overall variability between methods within the Hurricanes dataset remains small. For the biggest dataset, RaVAEn-Wildfires, variability is almost negligible, with all differences below 2 pp, meaning that all methods would effectively download nearly the entire dataset. For the smallest dataset, RaVAEn-Landslides, variability is the largest. Using NDVI for Landslides would save only  3.5 % of the downlink, whereas using the best model, a medium-sized STTORM-CD model, would save almost 54 %. It is thus obvious that dataset size plays a role. However, even for RaVAEn-Landslides, the difference between retrained STTORM-CD and the original RaVAEn is minor. For the medium and large models, STTORM-CD and RaVAEn alternate as the best method, with differences of less than 2 pp. For the small model, the difference is more pronounced, with RaVAEn outperforming STTORM-CD by  7.6 pp.

### Standard metrics

To place this research in the context of standard literature and to support the claims made using custom metrics, this section presents an evaluation using classical metrics. We show only two metrics here, AUPRC and F1 score, constructing the same tables as those for AURC and RDP to allow easy comparison while avoiding clutter in the article. Interested readers can find many more metrics, confusion matrices, and visualizations in the associated GitHub repository.

Unlike in RaVAEn, we compute the AUPRC, F1 score, and other metrics at the tile level rather than the pixel level, which is appropriate in this context. However, unlike the proposed AURC, both metrics require defining thresholds, which introduces a potential limitation for fair comparison across studies. The first threshold defines the minimum fraction of changed pixels in the ground truth needed for a tile to be considered changed. To remain consistent with RDP, this threshold is set at 5 % of pixels altered. For the F1 score here, as well as for other metrics in the associated repository, an additional threshold on the predicted change score, measured as cosine distance, is required. To ensure fairness across models and datasets, this threshold was set individually for each model and dataset, using the value that yielded the highest F1 score.

Results from the STTORM-CD-Floods test set are shown in Table 5. Both F1 and AUPRC exhibit similar trends, consistent with those observed using AURC, not in absolute values but in terms of relative method performance and interpretation. The STTORM-CD models perform best, while NDWI and the cosine baseline achieve similar results and rank second after STTORM-CD. RaVAEn performs the worst. The difference between STTORM-CD models and their RaVAEn counterpart is substantial, with  30–35 pp in AUPRC and  15–25 pp in F1 score. The best-performing STTORM-CD model is again the medium-sized one, and optimal results are obtained using *avg*(*memory*), although the distinction against *min*(*memory*) is negligible for both AUPRC and F1 score.

Table 6 shows results from the RaVAEn datasets. As before, the absolute values differ, but the interpretations and comparisons across methods are similar to those observed for AURC. STTORM-CD performs best for RaVAEn-Floods, outperforming its RaVAEn counterparts by  10 pp in AUPRC and  6–7 pp in F1 score. As with AURC, the loss or gain of STTORM-CD models relative to their RaVAEn counterparts is generally negligible to small for all model sizes and disasters, typically  2–4 pp loss in AUPRC and 0.5–2 pp in F1 score, except for Hurricanes, where the small model lost  12 pp in AUPRC and  11 pp in F1 score.

An interesting observation is the distinction between AUPRC and F1 score. The similarity between AURC and AUPRC is evident: for instance, in terms of AUPRC, the indices outperform the Cosine baseline by  3–10 pp across all datasets except RaVAEn-Floods, consistent with AURC results. However, when applying a single optimal threshold for the F1 score, these differences become negligible (0.5–2 pp), suggesting that the indices are stronger at ranking samples than at improving classification performance.

**Table 5:** Comparison of approaches’ performance on the test set from the STTORM-CD-Floods dataset via standard metrics. The “Most recent” column represents results from comparing the changed tile with only the most recent non-cloudy tile. In other columns, all non-cloudy tiles from memory were used to derive the final change predictions using the *min*() or *average*() function.

Method	AUPRC [%] $\uparrow$			F1 [%] $\uparrow$		
	Most recent	*min*(memory)	*avg*(memory)	Most recent	*min*(memory)	*avg*(memory)
Geo-Index	60.28	59.48	59.86	59.24	58.73	59.96
Cosine baseline	48.04	63.57	57.99	52.97	58.84	55.74
RaVAEn – small	39.21	43.92	**39.62**	46.90	50.57	48.15
RaVAEn – medium	**40.74**	**45.07**	39.60	**47.77**	**50.77**	**48.60**
RaVAEn – large	39.10	43.33	38.32	47.52	49.93	47.03
STTORM-CD – small	72.45	75.43	75.70	66.42	69.24	69.35
STTORM-CD – medium	**73.45**	**76.00**	**76.12**	**69.00**	**71.25**	**71.15**
STTORM-CD – large	69.05	72.31	72.26	63.18	66.70	66.67

Highlighted are the best scores for STTORM-CD models and RaVAEn models for easy comparison.

**Table 6:** Reevaluating RaVAEn. The final change predictions were derived by using $\min ()$ on the change predictions from the memory. In RaVAEn-Floods, the geo-index used was NDWI; in RaVAEn-Wildfires, the NBR; and in RaVAEn-Landslides and RaVAEn-Hurricanes, NDVI. Highlighted are the best scores for STTORM-CD and RaVAEn models for each metric. All values of the evaluation metrics are reported as percentages.

Dataset	RaVAEn-Landslides		RaVAEn-Wildfires		RaVAEn-Hurricanes		RaVAEn-Floods	
Tiles count	626		27865		11773		11253	
Changed tiles [%]	23.64		57.89		27.57		21.70	
Metric	AUPRC $\uparrow$	F1 $\uparrow$	AUPRC $\uparrow$	F1 $\uparrow$	AUPRC $\uparrow$	F1 $\uparrow$	AUPRC $\uparrow$	F1 $\uparrow$
Geo-index	71.69	67.92	95.16	88.60	79.12	72.27	52.53	50.76
Cosine baseline	68.06	67.48	92.52	87.40	69.55	70.57	65.63	70.30
RaVAEn – small	83.26	75.74	93.16	89.88	**75.87**	**67.06**	**70.92**	**66.91**
RaVAEn – medium	**84.72**	**75.91**	93.18	89.95	73.52	65.24	69.73	65.33
RaVAEn – large	83.12	74.68	**93.49**	**90.07**	71.85	63.52	68.11	64.86
STTORM-CD – small	80.72	**75.79**	90.46	89.30	63.88	56.88	81.67*	71.99*
STTORM-CD – medium	82.22	73.13	89.15	88.32	69.05	62.73	79.04*	72.02*
STTORM-CD – large	**82.60**	74.29	**93.42**	**89.33**	**70.75**	**64.36**	79.79*	71.84*

*Used as a validation dataset.

## Conclusion and future research

With the increasing impact of the climate crisis, more disasters like wildfires and floods will happen, and a disaster detection system could decrease the loss of lives and resources worldwide. This work lays the foundation for future research through the proposed metrics, baselines, and annotation strategy, while demonstrating the feasibility of constructing a universal and accurate disaster detection system. It aims to serve as an early step in global scientific collaboration, which can make this system a reality.

We demonstrated the potential for the production use of unsupervised VAE-based change detection due to its compression abilities. We also demonstrated how originally unpredictable results could be improved by fine-tuning via triplet loss and a relatively small dataset. The STTORM-CD models fine-tuned for floods scored clearly the best in AURC and AUPRC on the test set of STTORM-CD-Floods and the RaVAEn-Floods datasets. Aside from that, its performance on other disasters was only slightly affected. These results suggest that this methodology could be applied to a variety of onboard change detection tasks. Within the scope of disasters, it appears that a single model could potentially detect multiple types of disasters.

There was one exception, though: hurricanes. Here, a noticeable drop in performance was observed. We argue that this occurs because hurricane impacts are nearly impossible to distinguish from seasonal variations at coarse GSD, with only gradual vegetation loss visible, and damage to buildings and infrastructure not clearly seen. Therefore, hurricanes should be excluded from general disaster detection using traditional  10 m GSD imagery and instead classified among disasters detectable only with very-high-resolution imagery, such as earthquakes and conflicts, where structural damage is clearly visible. A more detailed visualization is provided in the Supplementary Information (see the “Hurricanes: Challenges with Coarse GSD” section, with Figure S4).

Besides that, it was also shown that the proposed baseline (geo-indices) performed quite well. Their compression ratios are maximized, and they were the best tool for delivering as much change as possible for hurricanes and wildfires when downloading only a limited part of the data (measured by AURC and AUPRC). Especially the NBR (Normalized Burn Ratio) performed quite well for the wildfires, suggesting that it could be a cheap machine-learning alternative to detect them. However, future studies should assess its performance on a larger, balanced dataset that includes raw onboard data, as defects of raw data could significantly impact accuracy and would require correction (such as coregistration of the misaligned bands), thereby increasing the processing time.

On the other hand, for classification tasks, its performance, measured by F1 score, is comparable to using the cosine distance of the flattened images, and if the goal is to download all changed data as quickly as possible, as measured by RDP, the performance of the geo-indices is inadequate. This is unsurprising as the margin between changed and unchanged regions can be quite small for geo-indices, as seen from the visualizations. Tiles with a small change proportion can then be easily mixed up with unchanged tiles. For these tasks, the STTORM-CD or RaVAEn models performed the best.

The indices could also be incorporated as additional inputs to the model, alongside other non-visual data such as location, Digital Elevation Models, or in-situ measurements (e.g., sea surface temperature), as explored in related studies^34–39^. However, the demands of generating, uplinking, or storing these products must be carefully considered, as the potential gain in accuracy may not always justify the additional requirements. The presented STTORM-CD models did not use the Sentinel-2 bands with coarse 60 m GSD, since these bands were not included in the pretraining of RaVAEn, even though two of them target water-related features such as coastal aerosol and water vapor. Despite this, the STTORM-CD models still performed well in flood detection. So, as an alternative direction, future studies could further reduce the number of input features, which may increase the system’s generalization while lowering both its requirements and the risk of overfitting. The system could operate with only RGB or RGB+NIR bands, as experimented with in another study^40^. Although this may reduce overall accuracy, it enables compatibility with low-cost smallsat cameras where the SWIR spectrum is often unavailable.

Regarding the metrics in general, the correctness of AURC was supported by standard metrics, while RDP remained distinct. Based on our observations, we do not recommend using RDP as a primary metric, as it is highly influenced by dataset size and distribution. However, as a complementary metric, it provides valuable additional information about potential bandwidth savings.

Another interesting finding is that the worst results were obtained for all datasets and metrics when the comparison was made only to the most recent picture. This suggests the importance of using multiple images of the area before for the change detection. This confirms the correctness of the storage-efficient approach and suggests the need for an even bigger compression ratio, which would enable the use of more images for change detection directly onboard the spacecraft. Future research could explore this by finding the optimal combination of input size, latent space size, and network architecture. Additionally, reducing the bit depth of latent space embeddings through half-precision floats or quantization could be investigated. An alternative way would be a memory management module, such as running average or LSTM, which would potentially enable keeping only one history-annuated embedding per area, ignoring clouds by itself and thus removing the need for a separate cloud detection algorithm.

However, a large, representative, and high-quality change detection dataset is needed for such future experiments, as the STTORM-CD-Floods dataset is too small to draw conclusive and reliable generalizations from the results. It should be worldwide and contain various disasters for accurate evaluation and effective training. Besides that, it should be balanced by a sufficient amount of time series containing only seasonal changes, such as blooming fields or snowing, so it would allow an accurate depiction of the method’s ability to ignore them. Ideally, it should be composed of data from multiple sensors. Sentinel-2 is an expensive, high-quality instrument, and we used 10 of its bands. It therefore remains unclear whether a model trained on it will perform well on cheaper, commercially viable sensors with different bands. Also, the raw data are needed as they contain various defects, and whether the model trained on processed data would work on the raw data available onboard is unclear. However, they are less available, so the onboard effects could be somewhat simulated.

The next step should be the development of onboard georeferencing, for which the current state overview is provided in the Supplementary Information (see the “Onboard georeferencing” section). Without georeferencing or a similar approach, it is impossible to connect the current picture embedding with the embedding of the same area from before. Also, it is not only needed for onboard change detection, but without it, it is quite hard to utilize the downlinked tile on Earth.

As the final step, it would be optimal to test and improve the system’s generalizability, ensuring that it performs accurately across various sensors and can be universally applied to multiple satellites. Once this is achieved, the system could be employed in an international mission, launching multiple satellites to monitor as large an area as possible. This would enable a short revisit rate, effectively creating an Earth surveillance network, similar to the approach proposed in^41^.

## Supplementary Information

Supplementary Information.

## Data Availability

We are releasing the training and test dataset on Zenodo^42^. The validation dataset and datasets focused on other disasters were released in the RaVAEn article^11^ and can be found on Google Drive, hosted by the RaVAEn authors. Additional visualizations, tables, source code, and models (including Apache TVM binaries for target processors) are all available on GitHub.
