# Supplementary material for: STTORM-CD low-demand and high-impact disaster monitoring onboard satellites using change detection
Source: Sci Rep. 2026 Feb 4;16:4939. doi: 10.1038/s41598-025-32598-3 (PMC12873372; doi:10.1038/s41598-025-32598-3)
Supplement: Supplementary file 1 — Supplementary Information. [file 41598_2025_32598_MOESM1_ESM.pdf]

STTORM-CD Supplementary Information

Jonáš Herec[1,2,*], Jan Sedmidubsky[1], and Rado Pitoňák[2]

[1]Masaryk University, Brno, Czech Republic
[2]Zaitra s.r.o., Brno, Czech Republic
[*]jonas.herec@zaitra.io

ABSTRACT

This supplementary information accompanies the STTORM-CD article. It provides a critical, in-depth assessment of existing tile-wise change detection evaluation methods, leading to the introduction of **two new metrics – Area under recall curve (AURC) and Required Downlink Percentage (RDP)**. This document also includes a survey on **onboard georeferencing**, an unsolved problem that is essential for effective onboard change detection. Additionally, a critique of including hurricanes for detection at coarse GSD is provided, along with an ablation study supporting the use of the variable margin, as well as further details regarding training.

Contents

1 Metrics **2**
1.1 RaVAEn evaluation and its problems . . . 2
1.2 Proposed primary metric – AURC . . . 3
1.3 Proposed secondary metric – RDP . . . 5
2 Onboard georeferencing **5**
3 Hurricanes: Challenges with Coarse GSD **6**
4 Training details and ablation study **7**

1 Metrics

This section will introduce the RaVAEn [1] evaluation method and show why it brings biased results. Following up, new metrics will be proposed.

1.1 RaVAEn evaluation and its problems

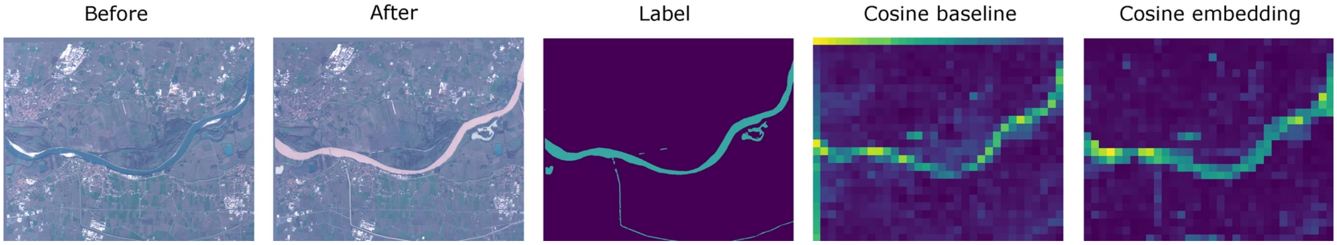

Figure S1. RaVAEn evaluation system. Heatmaps on the right, capturing the change predictions, are compared against the ground truth mask in the middle. Figure taken from RaVAEn article [1].

To evaluate their models, RaVAEn created heatmaps based on predicted cosine distances, as shown in Figure S1, and compared them with the ground truth masks. This means assigning a cosine distance value to each pixel in the heatmap. If a tile had a cosine distance of 0.4 compared to the reference tile, all pixels in that tile would be given a value of 0.4. This keeps the resolution of the heatmaps the same as the ground truth labels. These heatmaps were then checked against the ground truth masks using the Area under the Precision-Recall Curve (AUPRC). The Recall and Precision were defined as shown in Equations 1 and 2. For each disaster dataset (RaVAEn-Floods, RaVAEn-Wildfires, etc.), the reported statistic was the mean AUPRC, where the AUPRC was calculated for each time series individually, and then the mean was computed.

$$\text{Recall} = \frac{\text{Retrieved changed pixels}}{\text{All changed pixels}} \tag{1}$$

$$\text{Precision} = \frac{\text{Retrieved changed pixels}}{\text{Retrieved pixels}} \tag{2}$$

Using the area under the precision-recall curve is a good idea because it avoids setting a specific threshold for tile classification. This is particularly important in this task, where the focus is more on the order of tiles rather than strict classification, so assessing how well our model performs at various thresholds provides more information than selecting the best-performing threshold and computing the F1-score or similar metric. However, evaluating the AUPRC on a per-picture basis and then averaging is not ideal. This approach involves adjusting the threshold to change recall levels, which may not accurately reflect real-world conditions when applied individually to each picture. Instead, it should simultaneously be applied to all images in each dataset. This simulates real use more, where we would decide the best classification threshold beforehand, and it would stay the same for all the images taken.

But, in this specific case, relying on precision and recall impairs the score. Consider the results obtained for different disaster types: RaVAEn achieved the highest AUPRC of 0.913 for wildfires, while the lowest AUPRC of 0.448 was obtained for floods. When describing the dataset, it's noted that while a similar number of locations represent each type of event, the affected area varies significantly depending on the disaster type. Specifically, the RaVAEn-Wildfires dataset exhibits the largest area of effect and the largest proportion of changed pixels. This observation appears to be more than coincidental.

Wildfires tend to spread uniformly, consuming everything in their path. As a result, they often leave behind extensive burned areas, meaning that many tiles in the wildfire dataset have nearly all pixels marked as changed in the ground truth. In contrast, floods spread differently. Water flows into lower-altitude areas and may accumulate in certain spots while receding elsewhere. Consequently, the impacted area by floods is often more dispersed than that of wildfires. In many cases, the signs of a flood may not occupy the entire 32x32px area of a tile. This leads to floods producing numerous tiles containing flooded pixels to some degree, but rarely are all pixels in a tile marked as changed. If we apply RaVAEn's method to floods, it could significantly underestimate the model's performance. Even more important is that when comparing two models across the same disaster, the difference in performance is diminished by a priori given precision.

This impairment will be demonstrated through a fictitious example featuring two images: one of a wildfire and one of a flood. Each image contains 10 tiles with changes, with the wildfire tiles having 90 % of their pixels changed and the flood tiles having 30 % of their pixels changed. Two methods were used: a baseline model and a trained model. For simplicity, assume

that neither model makes any false positives, but they differ in their false negative rates. At the same threshold, the trained model correctly identifies 8 changed tiles, while the baseline identifies 2 changed tiles.

At this threshold, the recall for the baseline is 20 %, and for the trained model, it is 80 %, showing a significant improvement of the trained model over the baseline in both cases. However, when calculating the AUPRC in RaVAEn, this improvement is not accurately reflected. This is because precision is determined by the number of changed pixels in the tiles, which is 90 % for the wildfire tiles, and 30 % for the flood tiles (after the threshold). An illustration of this case can be seen in Figure S2. In the wildfire case, the AUPRC for the baseline is 0.91, while the trained model has an AUPRC of 0.94. For floods, the baseline's AUPRC is 0.37, and the trained model's AUPRC is 0.58. In both cases, the 60 % improvement is diminished by the precision, which can not be changed by the model, but it is given.

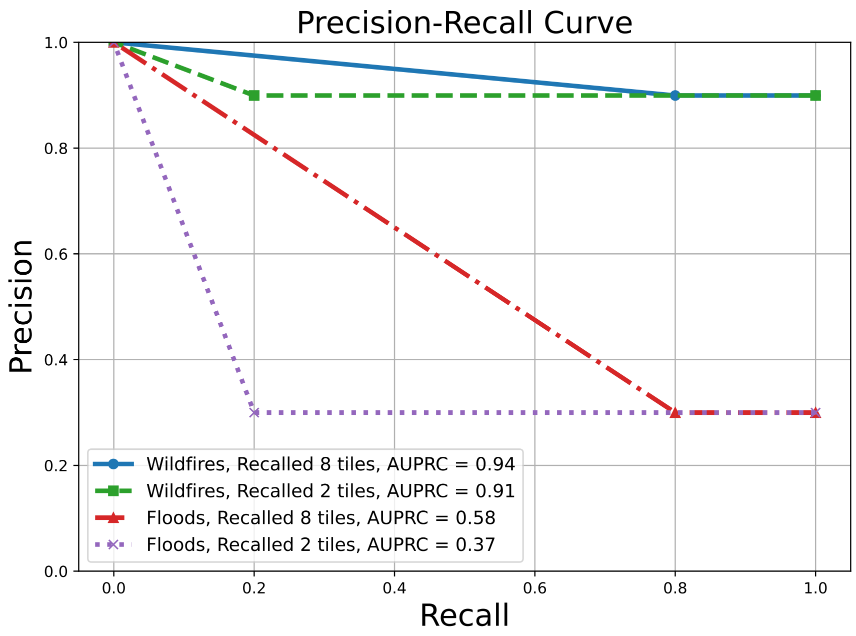

Figure S2. Illustration of RaVAEn evaluation method and its impairments. The same difference in model performance yields very different results across the disasters because of a given change distribution in the dataset.

As a result, the dataset structure heavily influences the metrics, failing to evaluate the model performance. The only thing that can be read from them is the binary assessment of whether the model performs better than the baseline on a given disaster.

1.2 Proposed primary metric – AURC

Here, an alternative approach will be proposed to address this impairment. As previously mentioned, the change detection system operates fundamentally as an ordering system. When a disaster occurs, we prioritize identifying the most severely affected areas, followed by the peripheries. One might argue that this resembles a regression problem, where we could measure the correlation between predicted change and the proportion of changed pixels. While this approach isn't entirely incorrect, it overlooks the essence of the task. Correlation indicates whether there's some degree of ordering present, but it doesn't provide insight into recall or precision. In simpler terms, it doesn't inform us about how many significant areas we've successfully identified versus those we've missed.

We instead propose another solution: the tiles will be ordered by predicted change, and only the upper portion of the predictions will be used to calculate recall on a per-pixel basis. For example, it could answer the question: If the top 10 % of tiles, when ordered by the predictions, will be retrieved, how many changed pixels will it retrieve?

For instance, if the top 10 % of the tiles ordered by ground truth contain 100 changed pixels, but the top 10 % of tiles, when ordered by the predictions, only contain 78 changed pixels, the recall score would be 78 %. This method focuses on the capability of the predictions to order the tiles, but does it more practically than correlation. Because it directly tells us how many pixels we have actually retrieved from those that could have been retrieved. However, this approach still presents biases. For instance, in the RaVAEn article [1], the flood dataset has 6.74 % of pixels classified as changed, while the wildfire dataset has 53.79 % of pixels classified as changed. Using a fixed threshold, e.g., top 10 % of tiles, might not make sense — this could unnecessarily include unchanged tiles for floods and too few changed tiles for wildfires, failing to provide informative insights about the remaining changed tiles.

That's why we have modified this method further. The recalled percentage will be calculated solely from changed tiles. For example, if a dataset contains 500 tiles in total but only 120 of them contain changed pixels, retrieving 100 % means retrieving the 120 most changed tiles when sorted by our change prediction, and retrieving 10 % means retrieving 12 tiles. This adjustment serves two purposes. Firstly, it ensures fairness by allowing a perfect system to retrieve all changed tiles and nothing more. Secondly, this approach maintains fairness regardless of the change distribution across tiles. If there are a lot of changed tiles with a big proportion of changed pixels, a fully changed tile will have a small influence on the result. Conversely, if there are only a few changed tiles and they contain only a small number of changed pixels, each tile will have a significant influence on the metric. This allows for an accurate comparison of the models within various datasets with different change distributions.

Moreover, in real-world usage, we might download a different portion of tiles than just 10 %. Therefore, arbitrarily selecting this percentage or any other value doesn't accurately reflect the practical use case. It could also become a testing "hyperparameter", which, instead of truly reflecting the model's performance, is being tweaked to achieve visually appealing results. That is why the final modification is calculating recall for all one hundred percent and then using the area under the curve to derive the final metric. This gives us an accurate depiction of model performance for downloading various amounts of data. This system is illustrated with a fictional example and larger steps between the retrieved thresholds in Figure S3.

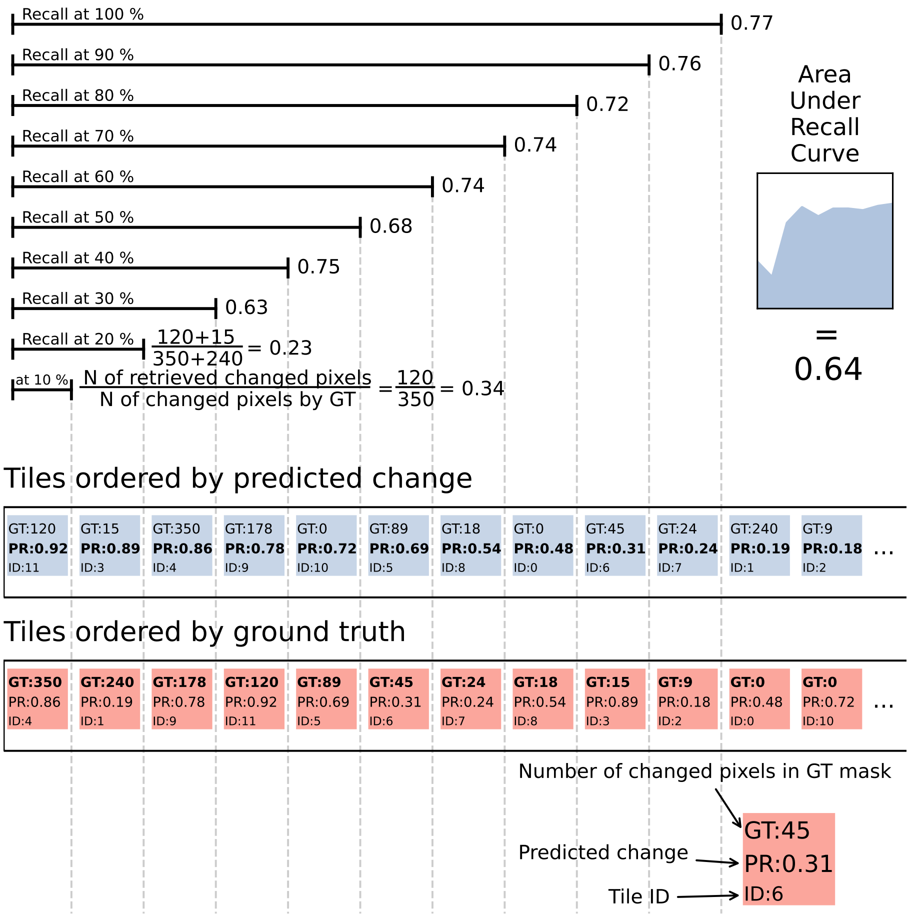

Figure S3. Proposed primary metric - AURC: The actual step size, which we use is 1 %, but for simplicity, it is illustrated with a step size of 10 % for a fictitious, very small dataset that contains only 10 changed tiles.

This approach ensures a fair comparison of methods within a single dataset while providing interpretability and accuracy that mirrors real-world scenarios. Some might argue that excluding precision from the evaluation is flawed, as it overlooks a comprehensive assessment of method efficacy. However, implicit precision is inherently embedded within this approach because including unchanged tiles diminishes the recall, and this effect is inversely proportional to the number of retrieved tiles. In top percentages, where almost all tiles are fully changed, including unchanged tiles can be costly. Inversely, when retrieving 100 % of changed tiles, the tiles at the bottom have only a few pixels changed, and including unchanged tiles is thus less costly. This is in sync with our use case, where the more changed tiles are more important.

Moreover, literal per-pixel precision holds less significance in this context. The primary objective is to identify and retrieve tiles with changes, prioritizing those with more significant changes, such as fully flooded areas, over partially flooded or unchanged areas. Hence, the emphasis lies more on the portion of change captured rather than the number of unchanged pixels spared. Additionally, considering that catastrophic events are infrequent, it's highly likely that satellite data used for disaster detection also serves other purposes. Therefore, downloading a tile with no change doesn't necessarily have negative implications as long as we've successfully captured most of the change.

However, despite its alignment with real-world applications, interpretability, and fair comparisons within a dataset, it's crucial to acknowledge that this approach doesn't guarantee equitable comparisons between different disaster types. The conditional probability of tiles within the changed region being changed remains higher for datasets with a greater change proportion (wildfires). However, this effect should diminish with larger and more balanced datasets, as well as with a more accurate change detection model, where randomness should play a smaller role. However, treating each disaster type as a separate dataset and comparing the distance between baseline and model performance, rather than solely comparing absolute metrics, is essential.

1.3 Proposed secondary metric – RDP

The proposed approach, however, can miss one important information. How much data do we need to downlink to receive all the changed tiles? This is particularly an issue when the size of the disaster differs. For example, in the case of a small island, the disaster could hit the entire island, which is quite important information. However, it does not have to be reflected in the proposed metric because only a few tiles have been changed. Such is the case of the RaVAEn-Hurricanes dataset [1]. It has one big event with many changed tiles and then smaller events. If the model performs well on the big event, the performance on the smaller events does not affect the metric significantly.

That is why a secondary metric will be used. It is very simple – it measures the amount of data needed to downlink to receive all changed tiles. For this, we must define what changed means, which is problematic. We are probably not interested in tiles containing only a few pixels of change. However, we don't want to miss even the small changes. So, we will use 5 % as the threshold. In the case of 32x32 px tiles, this is roughly 7x7 px, representing app. 70x70 m area. This allows us to focus on tiles with a small but meaningful change. When used with the primary metric, which focuses more on the top changed tiles and the sheer amount of change, it can complete the picture of the model's performance.

2 Onboard georeferencing

For the onboard change detection system to function, the current image needs to be matched quite accurately with an image from the past. To achieve this, some level of georeferencing is required. This remains an unsolved challenge, and surprisingly, none of the onboard change detection studies (cited in the main text) address this issue. The geolocation derived from the satellite's GPS is often insufficient for precise georeferencing. On Earth, this problem is typically resolved using indirect georeferencing, which involves aligning a georeferenced reference image with the raw image. However, storing such reference images onboard the satellite is not feasible due to the limited onboard storage space.

There are a few proposed solutions currently available. Most of them focus on specific parts of the georeferencing pipeline, but only [2] proposes a complete system. Their system suggests a robust, lightweight database of georeferenced features that can be matched via template matching (in this case, normalized cross-correlation) to the current raw image. First, they extract edges from the raw image using phase congruency, an edge extractor invariant to contrast and illumination, which produces standardized values between 0 and 1, making it ideal for use with various satellites and sensors. These values are then thresholded to reduce each pixel to a single bit. Run-length encoding compression is then applied to further reduce the image size. Only a subset of the image, focusing on invariant features like coastlines, roads, or airports, is stored, making the approach suitable for various satellites and sensors. This results in a lightweight database created from reference images of Earth. During flight, images are matched to reference images based on geographic position. The images are downsampled to reduce details and computational complexity before performing template matching. Harris corner points are detected, and the image is divided into patches around these points for even distribution. These patches are then matched with the reference image, with outliers removed using RANSAC. The accuracy is 100% with errors under 10m, 93% under 5m, and an average error of about 3m. Processing time on an Arm A57 CPU is 31s, 4x slower than on a 256 CUDA cores GPU (7.26s) for a 7920×1200 image, raising concerns about its feasibility for smallsats and commercial use.

Another approach is more obvious; image matching is often done through algorithms like SIFT, SURF, or BRIEF, which extract key points in the image, and each key point is described by a vector (descriptor). Then you can use Euclidean distance or kNN to match the key points from the images. This holds potential for on-board purposes, as, for example, BRIEF uses a 256-bit binary vector as the descriptor. Storing 1 key point per 1 km with latitude and longitude stored as 32-bit floats for each point would require approximately 10 GB for storing the whole Earth (excluding the oceans). However, the key point extraction algorithms and matching of the key points tend to be computationally heavy, so recent research has proposed their

acceleration by doing the computation on an FPGA. Mostly, the researchers focus on fast and robust SURF - [3, 4]. But SURF descriptors consist of floats, so it takes significantly more space than BRIEF. So for our purpose, the most interesting study is the [5] which uses FPGA acceleration and SURF as a keypoint extractor but uses BRIEF descriptors and BRIEF matching, which consists of fast bit-wise XOR operations. Extraction, description, and matching of two 512x512 images took 2.62ms with the proposed method. Its score is also acceptable, with precision fixed at 100 % recall is 0.70-0.82 (70-82 % of key points were matched) for rural areas and 0.3 for bare soil. This is acceptable as only a few key points are required to match the images. However, it was tested on images from the same sensor, so the potential problem with this approach is the generalization. To have a complete solution, you need to upload a database to the satellite beforehand, created from available worldwide imagery such as Sentinel 2 data, and this approach's ability to extract the same key points on pictures from different sensors is untested.

If the performance of classical algorithms is insufficient, another possible approach would be using a neural network for key points extraction, such as D2-net [6], which can robustly extract the same features and correctly match images. The input could be a panchromatic image or an RGB image, which would make it usable across most sensors. However, for its on-board use, it would need to be compressed or replaced by a smaller network, and it is unclear if this network would perform precisely and fast enough.

3 Hurricanes: Challenges with Coarse GSD

Wildfires, floods, and landslides are detectable even at the coarse 10m ground sampling distance (GSD) of Sentinel-2. In contrast, we argue that hurricanes are not. Figure S4 shows only one example from RaVAEn-Hurricanes, but the rest of the dataset exhibits similar characteristics. Except for a single case involving flooding caused by a hurricane, all examples correspond to islands where the post-disaster images show not structural damage but primarily a reduction in vegetation cover. Such changes can result from natural seasonal cycles as well as from the disaster itself.

Moreover, the effects of hurricanes on vegetation are not immediate. While vegetation may be damaged right after the event and lie on the ground, it can remain green for several days before visible decay occurs. This raises questions about the usefulness of the rapid delivery of such imagery. For these reasons, we consider hurricanes outside the scope of analysis for coarse-resolution imagery and relevant only for high-resolution data, where changes and damage to individual buildings and plants can be observed.

We also argue that this explains why our model, trained to ignore seasonal variations, exhibited the largest drop in performance for the hurricane cases. In addition to the example shown here, all hurricane samples and method performances can be viewed directly in the visualizations available in the associated GitHub repository.

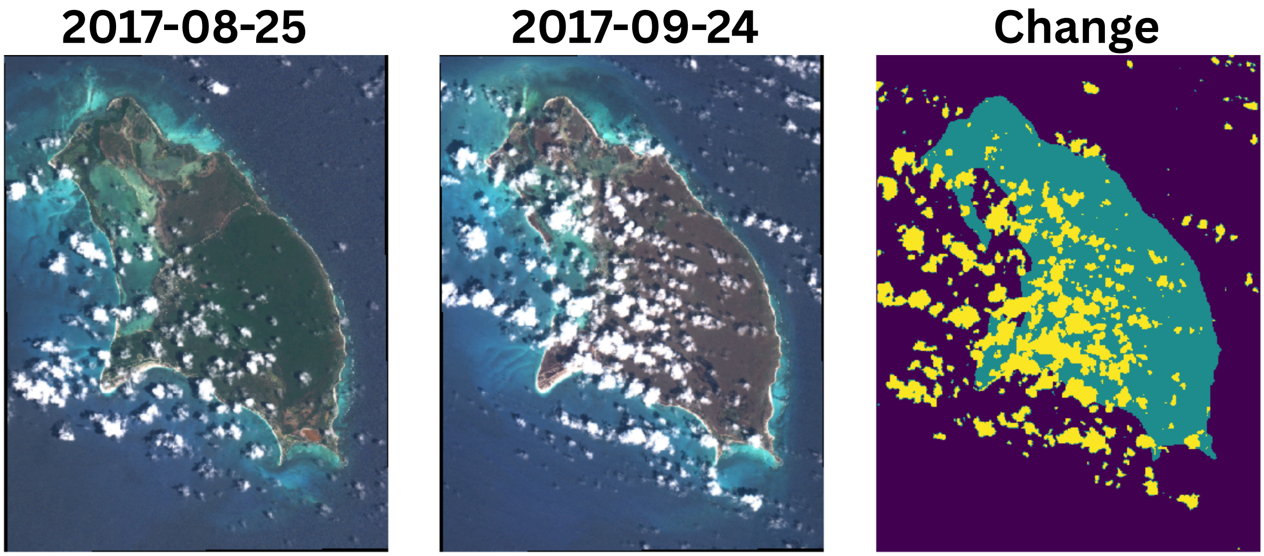

Figure S4. RaVAEn-Hurricanes – Hurricane Irma: The image shows the island of Barbuda. The last pre-disaster image is one month older than the post-disaster image, and the only visible difference is a reduction in vegetation, seen as the island turning brown. Under these conditions, it is challenging for the model to determine whether the observed changes are caused by the hurricane or by other natural processes.

4 Training details and ablation study

In this section, we use multiple metrics to compare margin strategies. For the classification metrics (AUPRC, RDP, F1, Precision, Recall), a tile was considered changed if more than 5% of its pixels were altered in the ground truth. For F1, Precision, and Recall, it is also necessary to define a threshold on the cosine distance to determine which predicted changes count as positive. To maintain fairness across models and datasets, the margin that yielded the highest F1 score for each model and dataset was selected for this purpose.

To identify the most effective configuration, we conducted an ablation study across our small, medium, and large models, comparing two margin strategies: **Variable** and **Fixed**. To remind the reader of the distinction: in the Variable strategy, the cosine distance between the changed and unchanged images is set equal to the fraction of changed pixels in each image, which allows the model to use both changed and unchanged triplets during training. In contrast, the Fixed strategy applies a constant margin (e.g., 0.5), which does not change and directly defines the minimum percentage of changed pixels required (e.g., a margin of 0.4 includes only triplets with at least 40% of pixels changed).

For each combination of model size and margin strategy, we selected the model that achieved the highest AURC on the validation dataset (RaVAEn-Floods), using the $avg(memory)$ configuration to derive change predictions. This approach ensured that performance on the target disaster (floods) was evaluated comprehensively, without relying on the test data.

The hyperparameters of the best-performing models are listed in Table S1, and all experiment runs are publicly available at Weights & Biases. The summarized results in Table S2 led us to adopt the Variable margin strategy for all model sizes.

Performance differences between margins were mostly small, though some were substantial (~5–10% or more). The effect also depended on the dataset: RaVAEn-Landslides showed pronounced differences, likely due to its small number of tiles. However, this is not universally true, as Hurricanes also exhibited larger performance gaps despite dataset sizes similar to or smaller than the Flood datasets.

For the small model, Variable margin was clearly better across nearly all metrics and datasets, with only 0–2 exceptional metrics per dataset. Specifically, RDP was worse by 6.8% for STTORM-CD-Floods, while other metrics were marginally better (~0.5–3.82%). Across other datasets, Recall/Precision trade-offs were observed, but F1 remained consistently higher with the Variable margin. On Hurricanes, Recall increased dramatically (25.31%) while Precision decreased only slightly (0.44%).

For the large model, performance was similar across the Flood datasets, but Variable margin clearly outperformed on the other disasters, making it the obvious choice.

For the medium model, results were mixed. Variable margin slightly outperformed Fixed margin on STTORM-CD Floods in most metrics by ~1–5% but experienced a significant drop in RDP (14.96%). On RaVAEn-Floods, it was slightly worse (~1–8%), mainly due to Recall/Precision trade-offs, while differences in key metrics (F1, AURC, AUPRC) were minor (~0.3–2.6%). On the other datasets, the models are fairly balanced, but to maintain consistency, and since Variable margin performed marginally better on the primary metric (AURC) for the primary dataset (STTORM-CD-Floods), the medium model was also selected with Variable margin.

Size	B	LR	E	WD	Margin	Stride
small (fixed margin)	32	3×10^{-6}	55	1.5×10^{-5}	Fixed, 0.5	32
small (variable margin)	64	5×10^{-7}	45	2.5×10^{-6}	Variable	16
medium (fixed margin)	32	3×10^{-6}	55	1.5×10^{-5}	Fixed, 0.5	32
medium (variable margin)	32	2×10^{-6}	40	1×10^{-5}	Variable	32
large (fixed margin)	64	5×10^{-7}	55	2.5×10^{-6}	Fixed, 0.5	16
large (variable margin)	32	1×10^{-6}	40	5×10^{-6}	Variable	32

Table S1. Hyperparameters for STTORM-CD models. B: batch size, LR: learning rate, E: epochs, WD: weight decay, Margin: triplet loss margin (Fixed or Variable), Stride: tiling stride.

Model	Tiles N: Changed [%]: Metric	626 23.64 RaVAEn Landslides	27865 57.89 RaVAEn Wildfires	11773 27.57 RaVAEn Hurricanes	11253 21.70 RaVAEn Floods	6678 22.34 STTORM-CD Floods
Small (Variable)	AURC [%] ↑	**82.81 (+7.35)**	**89.09 (+2.40)**	**69.79 (+5.84)**	85.68 (-1.61)	**86.11 (+0.80)**
	RDP [%] ↓	**55.27 (-5.27)**	**99.15 (-0.11)**	**98.52 (-1.45)**	**87.45 (-5.70)**	80.44 (+6.80)
	AUPRC [%] ↑	**80.72 (+9.27)**	**90.46 (+2.24)**	**63.88 (+9.98)**	**81.67 (+0.37)**	**75.70 (+2.74)**
	F1 [%] ↑	**75.79 (+4.32)**	**89.30 (+0.31)**	**56.88 (+8.79)**	**71.99 (+0.12)**	**69.35 (+2.39)**
	Precision [%] ↑	**70.59 (+9.63)**	**87.56 (+0.06)**	45.55 (-0.44)	70.54 (-6.35)	**66.02 (+3.82)**
	Recall [%] ↑	81.82 (-4.55)	**91.11 (+0.57)**	**75.70 (+25.31)**	**73.49 (+6.03)**	**73.03 (+0.52)**
Small (Fixed)	AURC [%] ↑	75.46 (-7.35)	86.69 (-2.40)	63.94 (-5.84)	**87.29 (+1.61)**	85.31 (-0.80)
	RDP [%] ↓	60.54 (+5.27)	99.26 (+0.11)	99.97 (+1.45)	93.15 (+5.70)	**73.64 (-6.80)**
	AUPRC [%] ↑	71.46 (-9.27)	88.22 (-2.24)	53.90 (-9.98)	81.30 (-0.37)	72.96 (-2.74)
	F1 [%] ↑	71.47 (-4.32)	88.99 (-0.31)	48.09 (-8.79)	71.87 (-0.12)	66.96 (-2.39)
	Precision [%] ↑	60.96 (-9.63)	87.49 (-0.06)	**45.99 (+0.44)**	**76.90 (+6.35)**	62.19 (-3.82)
	Recall [%] ↑	**86.36 (+4.55)**	90.54 (-0.57)	50.39 (-25.31)	67.46 (-6.03)	72.52 (-0.52)
Medium (Variable)	AURC [%] ↑	**86.71 (+1.12)**	87.97 (-1.32)	**73.51 (+2.96)**	85.46 (-0.30)	**86.54 (+1.34)**
	RDP [%] ↓	**46.17 (-12.94)**	98.61 (+0.16)	**96.49 (-0.48)**	**89.31 (-0.25)**	88.26 (+14.96)
	AUPRC [%] ↑	**82.22 (+1.15)**	89.15 (-0.67)	**69.05 (+2.84)**	79.04 (-2.60)	**76.12 (+2.35)**
	F1 [%] ↑	73.13 (-0.77)	**88.32 (+1.64)**	**62.73 (+1.76)**	72.02 (-1.03)	**71.15 (+4.10)**
	Precision [%] ↑	**87.37 (+20.50)**	88.06 (-0.54)	**55.32 (+3.11)**	72.42 (-7.83)	**66.64 (+3.10)**
	Recall [%] ↑	62.88 (-19.70)	**88.58 (+3.75)**	72.42 (-0.82)	**71.63 (+4.59)**	**76.32 (+5.36)**
Medium (Fixed)	AURC [%] ↑	85.59 (-1.12)	**89.29 (+1.32)**	70.55 (-2.96)	**85.76 (+0.30)**	85.20 (-1.34)
	RDP [%] ↓	59.11 (+12.94)	**98.45 (-0.16)**	96.97 (+0.48)	89.56 (+0.25)	**73.30 (-14.96)**
	AUPRC [%] ↑	81.06 (-1.15)	**89.82 (+0.67)**	66.21 (-2.84)	**81.64 (+2.60)**	73.77 (-2.35)
	F1 [%] ↑	**73.90 (+0.77)**	86.68 (-1.64)	60.97 (-1.76)	**73.05 (+1.03)**	67.05 (-4.10)
	Precision [%] ↑	66.87 (-20.50)	**88.60 (+0.54)**	52.21 (-3.11)	**80.25 (+7.83)**	63.54 (-3.10)
	Recall [%] ↑	**82.58 (+19.70)**	84.84 (-3.75)	**73.24 (+0.82)**	67.04 (-4.59)	70.96 (-5.36)
Large (Variable)	AURC [%] ↑	**87.74 (+12.87)**	**92.38 (+6.95)**	**74.73 (+8.90)**	**84.50 (+0.16)**	**83.99 (+0.04)**
	RDP [%] ↓	52.08 (+0.80)	99.47 (+0.35)	**94.89 (-3.43)**	95.19 (+4.08)	**76.43 (-1.05)**
	AUPRC [%] ↑	**82.60 (+6.77)**	**93.42 (+6.72)**	**70.75 (+12.71)**	79.79 (-1.11)	72.26 (-1.32)
	F1 [%] ↑	74.29 (-2.01)	**89.33 (+3.51)**	**64.36 (+12.54)**	71.84 (-1.22)	66.67 (-0.37)
	Precision [%] ↑	70.27 (-4.37)	**89.52 (+4.45)**	**53.25 (+7.37)**	76.44 (-7.35)	61.57 (-4.19)
	Recall [%] ↑	**78.79 (+0.76)**	**89.15 (+2.57)**	**81.31 (+21.81)**	**67.77 (+2.99)**	**72.69 (+4.32)**
Large (Fixed)	AURC [%] ↑	74.87 (-12.87)	85.43 (-6.95)	65.82 (-8.90)	84.34 (-0.16)	83.94 (-0.04)
	RDP [%] ↓	**51.28 (-0.80)**	**99.12 (-0.35)**	98.32 (+3.43)	**91.11 (-4.08)**	77.48 (+1.05)
	AUPRC [%] ↑	75.83 (-6.77)	86.71 (-6.72)	58.04 (-12.71)	**80.90 (+1.11)**	**73.57 (+1.32)**
	F1 [%] ↑	**76.30 (+2.01)**	85.82 (-3.51)	51.81 (-12.54)	**73.07 (+1.22)**	**67.03 (+0.37)**
	Precision [%] ↑	**74.64 (+4.37)**	85.07 (-4.45)	45.88 (-7.37)	**83.79 (+7.35)**	**65.75 (+4.19)**
	Recall [%] ↑	78.03 (-0.76)	86.58 (-2.57)	59.50 (-21.81)	64.78 (-2.99)	68.37 (-4.32)

Table S2. Ablation study for margin types. Numbers in parentheses indicate the difference relative to the same model with the opposite margin strategy. Bold numbers highlight better results across margins for the same model size. For flood datasets (STTORM-CD Floods and RaVAEn Floods), the final change predictions were derived using $avg()$ on the memory. For the other RaVAEn datasets, we used $min()$ to filter out noise that the model was not trained for.

References

1. Ruzicka, V. *et al.* Ravaen: unsupervised change detection of extreme events using ml on-board satellites. *Sci. reports* **12**, 16939 (2022).

2. Wang, L., Xiang, Y., Wang, Z., You, H. & Hu, Y. On-board geometric rectification for micro-satellite based on lightweight feature database. *Remote. Sens.* **15**, DOI: 10.3390/rs15225333 (2023).

3. Yang, Z., Hu, C. & Liu, D. Fpga image stitching design based on improved surf algorithm. In *2022 International Conference on Artificial Intelligence and Computer Information Technology (AICIT)*, 1–4, DOI: 10.1109/AICIT55386.2022.9930281 (2022).

4. Du, X. *et al.* Hardware-optimized architecture of on-board registration for remote-sensing images —take surf as an example. *IEEE J. Sel. Top. Appl. Earth Obs. Remote. Sens.* **17**, 8230–8249, DOI: 10.1109/JSTARS.2024.3377663 (2024).

5. Liu, D., Zhou, G., Zhang, D., Zhou, X. & Li, C. Ground control point automatic extraction for spaceborne georeferencing based on fpga. *IEEE J. Sel. Top. Appl. Earth Obs. Remote. Sens.* **13**, 3350–3366, DOI: 10.1109/JSTARS.2020.2998838 (2020).

6. Dusmanu, M. *et al.* D2-net: A trainable cnn for joint detection and description of local features (2019). 1905.03561.
